# Common variants in the *hERG* (*KCNH2*) voltage-gated potassium channel are associated with altered fasting and glucose-stimulated plasma incretin and glucagon responses

**DOI:** 10.1186/s12863-018-0602-2

**Published:** 2018-03-16

**Authors:** Line Engelbrechtsen, Yuvaraj Mahendran, Anna Jonsson, Anette Prior Gjesing, Peter E. Weeke, Marit E. Jørgensen, Kristine Færch, Daniel R. Witte, Jens J. Holst, Torben Jørgensen, Niels Grarup, Oluf Pedersen, Henrik Vestergaard, Signe Torekov, Jørgen K. Kanters, Torben Hansen

**Affiliations:** 1 Novo Nordisk Foundation Center for Basic Metabolic Research, Section of Metabolic Genetics, University of Copenhagen, Faculty of Health and Medical Sciences, Blegdamsvej 3B, Maersk Tower 8. floor, -2200 Copenhagen, DK Denmark; 2 Danish Diabetes Academy, Odense, Denmark; 3 Department of Biomedical Sciences, Faculty of Health and Medical Sciences, University of Copenhagen, Copenhagen, Denmark; 4 Department of Cardiology, Rigshospitalet, Copenhagen, Denmark; 5 National Institute of Public Health, University of Southern Denmark, Odense, Denmark; 6 Steno Diabetes Center, Gentofte, Denmark; 7 Section of General Practice, Department of Public Health, Aarhus University, Aarhus, Denmark; 8 Research Centre for Prevention and Health, The Capital Region of Denmark, Hillerød, Denmark; 9 Department of Public health, Faculty of Health Science, University of Copenhagen, Copenhagen, Denmark; 10 Faculty of Medicine, Aalborg University, Aalborg, Denmark

**Keywords:** hERG ion channel, QT interval, *KCHN2*, Glucagon, Glucose-dependent insulinotropic polypeptide (GIP), Glucagon-like peptide-1 (GLP-1), Insulin, Genetic risk score

## Abstract

**Background:**

Patients with long QT syndrome due to rare loss-of-function mutations in the human ether-á-go-go-related gene (*hERG)* have prolonged QT interval, risk of arrhythmias, increased secretion of insulin and incretins and impaired glucagon response to hypoglycemia. This is caused by a dysfunctional Kv11.1 voltage-gated potassium channel. Based on these findings in patients with rare variants in *hERG*, we hypothesized that common variants in *hERG* may also lead to alterations in glucose homeostasis. Subsequently, we aimed to evaluate the effect of two common gain-of-function variants in *hERG* (rs36210421 and rs1805123) on QT interval and plasma levels of glucagon-like peptide-1 (GLP-1), glucose-dependent insulinotropic polypeptide (GIP), insulin and glucagon during an oral glucose tolerance test (OGTT).

We used two population-based cohorts for evaluation of the effect of common variants in hERG on QT-interval and circulation levels of incretins, insulin and glucagon. The Danish population-based Inter99 cohort (*n* = 5895) was used to assess the effect of common variants on QT-interval. The Danish ADDITION-PRO cohort was used (*n* = 1329) to study genetic associations with levels of GLP-1, GIP, insulin and glucagon during an OGTT.

**Results:**

Carriers of either the minor A-allele of rs36210421 or the minor G-allele of rs1805123 had ~ 2 ms shorter QT interval per risk allele (*p* = 0.025 and *p* = 1.9 × 10^− 7^). Additionally, both variants were associated with alterations in pancreatic and gut hormone release among carriers. The minor A- allele of rs36210421 was associated with increased GLP-1 and decreased GIP response to oral glucose stimulation, whereas the minor G-allele of rs1805123 is associated with decreased fasting plasma insulin and glucagon release. A genetic risk score combining the two gene variants revealed reductions in glucose-stimulated GIP, as well as suppressed glucagon response to increased glucose levels during an OGTT.

**Conclusions:**

Two common missense polymorphisms of the Kv11.1 voltage-gated hERG potassium channel are associated with alterations in circulating levels of GIP and glucagon, suggesting that hERG potassium channels play a role in fasting and glucose-stimulated release of GIP and glucagon.

**Trial registration:**

ClinicalTrials.gov (NCT00289237). Trial retrospectively registered at February 9, 2006. Studies were approved by the Ethical Committee of the Central Denmark Region (journal no. 20080229) and by the Copenhagen County Ethical Committee (KA 98155).

**Electronic supplementary material:**

The online version of this article (10.1186/s12863-018-0602-2) contains supplementary material, which is available to authorized users.

## Background

Loss-of-function mutations in the human ether-á-go-go related (*hERG)* gene result in a dysfunctional Kv11.1 voltage-gated potassium channel causing delayed cardiac repolarization, and long QT syndrome (LQTS) with increased risk of cardiac arrhythmias and sudden death [1–4]. LQTS is characterized by a prolongation of the QT-interval. The QT-interval is the time between the start of the Q-wave and the end of the T-wave in the ECG. The QT-interval reflects the depolarization and repolarization of the ventricles in the heart. Aside from cardiac muscle cells, voltage-gated *hERG*-encoded Kv11.1 potassium channels are expressed in a number of tissues throughout the body including endocrine intestinal K- and L-cells and pancreatic α- and β-cells [5–8]. This has led to speculations that hERG channels may play a role in secretion of hormones from endocrine cells [9].

Glucose homeostasis is tightly regulated by interaction between glucose-regulating hormones from endocrine intestinal cells and pancreatic islet cells. A well-regulated interplay between the incretins, glucagon-like peptide-1 (GLP-1) and gastric inhibitory polypeptide (GIP) from intestinal L- and K-cells, and the secretion of insulin and glucagon from the pancreatic islets ensures glucose homeostasis by limiting glucose excursions, by facilitating uptake and storage of glucose during fed states, and by regulating glucose production for glucose-dependent tissues during fasting conditions. Secretion from the pancreatic islets cells and L-cells is determined by their electrical activity which, among others, is regulated by different types of voltage-gated potassium channels, and the hERG channel is involved in repolarization of the membrane potential [6–12].

Recently, *Hyltén-Cavallius* et al. [9] reported that patients with LQTS due to impaired function of the *hERG*-encoded Kv11.1 potassium channel not only have alterations in their cardiac conduction, but also exhibit increased insulin, GLP-1 and GIP secretion with risk of hypoglycemia, as well as decreased fasting and hyperglycemia induced levels of glucagon. Based on the findings in carriers of rare variants, we hypothesized that common variants may also cause alterations in QT-interval and in circulating levels of incretins, insulin and glucagon levels. We selected non-synonymous coding variants with a minor allele frequency > 3% in *hERG* (rs36210421 and rs1805123). We used data from two large cohorts; the Inter99 cohort (*n* = 5487) to evaluate the effect of common variants in *hERG* on QT-interval and the ADDITION-PRO cohort (*n* = 1329) to evaluate the effect on common variants on circulating plasma levels of incretins, insulin and glucagon.

## Methods

### Populations

ADDITION-PRO is a cohort study of individuals at low to high risk of type 2 diabetes, nested within the population-based ADDITION-Denmark [13]. Overall, 1329 individuals were included - 708 normal glucose tolerance (NGT); 254 had isolated impaired fasting glucose (IFG); 103 had isolated impaired glucose tolerance (IGT); 116 had both IFG and IGT, and 148 had screen detected type 2 diabetes. Participants (48% females) had a mean age of 66.3 ± 6.9 and a mean BMI of 27.1 ± 4.6. All individuals who fasted < 8 h before the blood samples were taken, or who used diabetes medication, or individuals with already known diabetes were excluded (*n* = 22). Characteristics of the ADDITION-PRO participants included in the present study are given in Additional file 1: Table S1.

Inter99 is a population-based randomized intervention study of 6784 participants aiming at preventing ischemic heart disease by non-pharmacologic intervention (ClinicalTrials.gov, NCT00289237). The Inter99 study has been described in details previously [14]. We included 5487 individuals (51%) with a mean age of 46.2 ± 7.9 and a BMI of 26.2 ± 4.5. More characteristics on the study participants can be found in the Additional file 1: Table S1.

The ADDITION-PRO study was approved by the Ethical Committee of the Central Denmark Region (journal no. 20080229) and the Inter99 study was approved by the Copenhagen County Ethical Committee (KA 98155) and the National Board of Health. Both studies were conducted in accordance with the principles of the Helsinki Declaration. All study participants provided written informed consent.

### Biochemical measures

#### ADDITION-PRO and Inter99

Baseline venous blood samples were drawn after an overnight fast (≥8 h). Participants underwent a standard 75 g oral glucose tolerance test (OGTT) with blood samples drawn at 0, 30 and 120 min. Measurements of circulating levels of glucose, insulin, GLP-1, GIP, and glucagon were collected during the OGTT. Serum insulin was measured by immunoassay (AutoDELFIA, Perkin Elmer, Massachusetts, United States). Plasma glucose was measured using the Hitachi 912 system (Roche Diagnostics, Mannheim, Germany) or the Vitros 5600 system (Ortho Clinical Diagnostics, Illkirch Cedex, France). Blood samples for the measurement of GLP-1, GIP and glucagon were obtained in tubes containing EDTA and put on ice immediately, before centrifugation at 4 °C. Plasma was stored at − 80 °C. Total plasma GLP-1 (intact GLP-1 plus the metabolite GLP-1 (9–36) amide), total plasma GIP (the sum of intact GIP plus the metabolite GIP 3–42) and plasma glucagon were determined using radio-immunological assays, as previously described [15–17].

### Electrocardiogram measures (ECG)

All ECGs were obtained on inclusion into the Inter99 cohort and were digitally recorded and stored in the MUSE Cardiology Information System (GE Healthcare, Wauwatosa, WI, USA) and processed using Marquette 12SL algorithm, version 21. ECG measurements were assessed and analyzed digitally for heart rate and mean QTc interval. QT- interval was used for assessment of the impact of genetic variants on cardiac conduction. QT interval was corrected for heart rate using Fridericias formula (QTcF = QT / ^3^√RR) [18]. The RR interval refers to the time between R waves.

### Genetics

Non-synonymous coding variants in *hERG* were included in this study. The variants were missense variants in *hERG* with a minor allele frequency > 3% causing amino acid substitutions with a significant effect on the *hERG* channel function [19–21]. Two variants were chosen, rs36210421 and rs1805123. Previous functional in vitro and in vivo studies [19–21] and population-based studies [20–25] have demonstrated that the minor A-allele of rs36210421 (R1047L) and minor G-allele of rs1805123 (K897 T) have gain-of-function effects on the hERG channel.

#### Addition-pro

We genotyped 1657 participants of the ADDITION-PRO cohort applying the Illumina Infinium HumanCoreExome Beadchip (Illumina, San Diego, CA). We excluded individuals which were first degree relatives, duplicates, ethnic outliers, or individuals who had extreme inbreeding coefficients, mislabeled gender, or call rate < 95%, leaving 1342 individuals who passed quality control criteria [26]. A total of 1329 individuals had information on GLP1, GIP, glucagon, fasting glucose and fasting serum insulin levels. Additional genotypes were imputed (rs36210421) with high quality (proper_info > 0.95) into the 1000 genomes phase 1 panel using IMPUTE2. All variants were in Hardy Weinberg equilibrium (*P* > 0.05) [27].

#### INTER-99

In the Inter99 cohort, 6161 individuals were genotyped with the Illumina Human Exome BeadChip v1.0 genotyping array (“the exome chip”), as previously described [28].

### Statistical analyses

Statistical analyses were performed using R, version 3.1.3 (https://www.r-project.org/). The trapezoidal method was used for calculation of total area under the curve (AUC), incremental area under the curve (iAUC) for GLP-1, GIP, insulin and glucagon. Calculations can be found in Additional file 1. We used multiple linear regression analysis to evaluate the association of QTcF, fasting and stimulated levels of glucose, insulin, GLP-1, GIP, and glucagon (as dependent variables) with the minor A-allele of rs36210421 and the minor G-allele of rs1805123 SNPs/ GRS (as independent variable). Effect sizes (β coefficients) per copy of the risk alleles of the SNPs investigated were estimated by linear regression analysis adjusted for age, sex and BMI. In order to adjust for population stratification, relatedness and to limit the type 1 error we calculated genome-wide principal components (PC) and adjusted our model for the first three PCs along with other covariates. *P* values < 0.05 were considered significant.

#### Genetic risk score (GRS)

To evaluate the combined effect of the two gene variants, rs36210421 and rs1805123, on QT-interval and metabolic phenotypes, an additive model was used to construct an unweighted genetic risk score. The GRS was calculated by summation of the number of risk alleles across the two gene variants.

## Results

The variants rs36210421 and rs1805123 in *hERG* were present in individuals from the ADDITION-PRO cohort with a minor allele frequency (MAF) of 3.4% (A-allele) and 24% (G-allele) respectively, and in Inter99 with a MAF of 3.1% and 22%, respectively. We found a low degree of linkage disequilibrium between rs36210421 and rs1805123 (R^2^ = 0.005 and D prime = 0.241). Demographic description of the Inter99 and the ADDITION-PRO cohort can be seen in Additional file 1: Table S1.

### Association of gene variants with duration of QT interval

The minor A-allele of rs36210421 (R1047L) and the minor G-allele of rs1805123 (K897 T) were significantly associated with shorter QTcF interval (β = − 2.4 ms, *p* = 0.016 and β = − 2.1 ms, *p* = 1.3 × 10^− 7^) in the Inter99 study (Table 1).

**Table 1:** Association of variants (rs36210421 and rs1805123) with QT interval and metabolic changes in INTER99 cohort (*n* = 5487)

	rs36210421 (MAF = 0.031) C > A		rs1805123 (MAF = 0.22) T > G		GRS	
Trait	β (95% CI)	*P*	β (95% CI)	*P*	β (95% CI)	*P*
QTcF (ms)	− 2.4 (− 4.31 – (− 0.47))	**0.016**	−2.12 (− 2.91 – (− 1.33)	**1.3E-07**	−2.3 (− 3.05– (− 1.54)	**2.2e-9**
Fasting Glucose (mmol/L)	0.06 (− 0.02 – 0.14)	0.12	−0.01 (− 0.05–0.02)	0.84	−0.002 (− 0.03–0.03)	0.42
Glucose 30 min (mmol/L)	0.11 (− 0.07–0.29)	0.32	0.007 (− 0.07–0.08)	0.66	0.02 (−0.05–0.095)	0.43
Glucose 120 min (mmol/L)	0.05 (− 0.17–0.26)	0.76	−0.06 (− 0.15–0.03)	0.73	−0.05 (− 0.14–0.035)	0.88
Fasting Insulin (pmol/L)	0.57 (−2.07–3.22)	0.60	0.34 (− 0.75–1.44)	0.87	0.40 (− 0.64–1.45)	0.73
Insulin 30 min (pmol/L)	10.41 (− 7.90–28.72)	0.12	0.91 (− 6.67–8.49)	0.82	2.23 (− 4.77–9.67)	0.41
Insulin 120 min (pmol/L)	5.21 (− 15.8–26.22)	0.34	−6.86 (− 15.53–1.81)	0.32	−5.44 (− 13.71–2.84)	0.57

QTcF interval measured in milliseconds. β and *P* values were adjusted for age, sex and BMI. B and 95% is the effect size estimate using untransformed values. The *P* values were obtained from the inverse normal transformation. GRS is the additive effect of both SNPs on QT interval. β, 95% and *P* values adjusted for age, sex and BMI. *P* values < 0.05 are in bold

### Associations of gene variants with fasting and stimulated levels of glucose, insulin, GLP-1, GIP, and glucagon

In the ADDITION-PRO cohort the minor G-allele of rs1805123 (K897 T) was significantly associated with 3.18 pmol/L (CI -5.87;-0.49)) lower fasting insulin (*p* = 0.025). The minor G-allele was associated with 0.95 pmol/L (− 1.52; − 0.38)) lower fasting glucagon (*p* = 0.003) (Additional file 1: Table S3). Additionally, we found decreased glucagon AUC at 0–30 and 0–120 min (β = − 2.73 (− 4.73, − 0.70), *p* = 0.009 and β = − 2.27 (− 4.25, − 0.24), *p* = 0.029, respectively) (Additional file 1: Table S3). The minor A-allele of rs36210421 was associated with decreased fasting GIP (*p* = 0.04) and decreased GIP_120min_ (p = 0.04), as well as increased GLP-1 iAUC at 30 and 120 min (p = 0.02 and p = 0.04). In Inter99, plasma glucose and serum insulin levels were not associated with the two gene variants (Table 1).

**Fig. 1:**
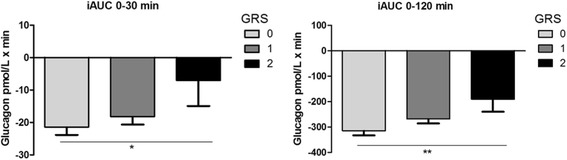
Incremental glucagon levels according to number of risk alleles. Mean and SEM of decremental glucagon levels according to genetic risk score (GRS) from (rs36210421 and rs1805123). GRS = 0 indicates no risk alleles, GRS = 1 indicates carriers of one risk allele of either rs1805123 or rs36210421, GRS = 2 indicates two or more risk alleles of either rs1805123 or rs36210421. *P* values indicate statistical significance, obtained from multiple linear regression adjusted for age, sex and bmi. *P < 0.05; **P < 0.01

We calculated an unweighted genetic risk score (GRS), assessing the additive effect of the minor A-allele of rs36210421 and the minor G-allele of rs1805123 on metabolic measures (Table 2, Fig. 1 and Additional file 1: Table S2, and Fig. S2). The GRS was significantly associated with lower fasting insulin (β = − 3.77 (− 6.36, − 1.18), *p* = 0.004), lower fasting GIP (β = − 0.60 (− 1.16, − 0.04), *p* = 0.041) and lower fasting glucagon (β = − 1.08 (− 1.78, − 0.38), *p* = 0.005). Additionally, the GRS was significantly associated with GIP AUC at 0–30 min was significantly decreased (β = − 1.96 (− 4.10, − 0.19), *p* = 0.044).The GRS was further associated with a decrease in glucagon AUC at 0–30 min (β = − 2.27 (− 4.21, − 0.29), *p* = 0.025), although not significant at 0–120 min (β = − 1.85 (− 3.78, 0.12, *p* = 0.066). Additionally, there was higher decremental glucagon AUC at 0–30 min, *p* = 0.011 and at 0–120 min, *p* = 0.003). Changes in plasma levels of glucagon during an OGTT according to number of risk alleles are shown in Fig. 1 and Additional file 1: Figure S2.

**Table 2:** Association of unweighted genetic risk score with metabolic and incretin levels in ADDTION-PRO cohort (*N* = 1324)

Trait	Median (inter- quartile range)			Beta (95% CI)	*P*
GRS (n)	0 (*n* = 708)	1(*n* = 523)	2(*n* = 93)	–	–
Gender (male/female)	375/333	269/254	48/45	−0.01 (− 0.05, 0.04)	0.222
Age, years	67.15(62.35–72.17)	65.94 (61.64–70.96)	66.12 (61.69–70.75)	− 0.79 (− 1.39, − 0.19)	0.005
BMI, kg/m^2^	26.88 (24.16–29.94)	26.35 (23.86–29.23)	27.73 (25.17–30.05)	− 0.16 (− 0.55, 0.24)	0.443
Fasting glucose (mmol/L)	5.90 (5.56–6.40)	5.90 (5.56–6.37)	5.97 (5.56–6.47)	− 0.00 (− 0.07, 0.06)	0.848
Glucose 30 min (mmol/L)	9.22 (8.10–10.30)	9.02 (7.99–10.10)	9.12 (8.10–10.03)	− 0.09 (− 0.24, 0.05)	0.251
Glucose 120 min (mmol/L)	6.47 (5.25–7.99)	6.24 (5.25–7.60)	6.40 (5.20–7.70)	−0.07 (− 0.26, 0.12)	0.818
Glucose AUC_30min_ (mmol/L x min)	227.78 (205.50–247.6205)	224.73 (204.89–243.04)	226.26 (206.42–246.09)	− 1.35 (− 4.23, 1.53)	0.424
Glucose AUC_120min_ (mmol/L x min)	924.43 (828.73–1054.57)	915.71 (822.63–1030.16)	930.97 (818.05–1050.00)	−8.32 (− 23.67, 7.03)	0.364
Glucose iAUC_30min_ (mmol/L x min)	47.30 (36.62–61.04)	45.00 (33.00–59.51)	44.25 (35.09–56.46)	− 1.38 (− 3.11, 0.36)	0.123
Glucose iAUC_120min_ (mmol/L x min)	221.26 (141.00–306.71)	205.50 (129.00–296.03)	212.11 (131.23–306.00)	−8.14 (− 19.59, 3.32)	0.206
Fasting Insulin (pmol/L)	39 (25–59)	35 (25–50)	38 (23–58)	− 3.77 (− 6.36, − 1.18)	0.004
Insulin 30 min (pmol/L)	223 (153–334)	213 (144.5–292.5)	227 (144–335)	− 8.16 (− 23.59, 7.27)	0.255
Insulin 120 min (pmol/L)	190 (115–328.5)	175 (105.5–299.5)	211 (112–294)	− 5.05 (− 26.61, 16.51)	0.460
Insulin AUC_30min_ (pmol/L x min)	4020.0 (2722.5–5842.5)	3750.0 (2602.5–5197.5)	3930 (2535–5790)	− 173.04 (− 422.51, 76.43)	0.152
Insulin AUC_120min_ (pmol/L x min)	23,535.0 (16,252.5–33,592.5)	21,832.5 (14,985.0–32,415.0)	22,080.0 (17,055.0–37,785.0)	− 727.72 (− 2335.91, 880.47)	0.202
Insulin iAUC_30min_ (pmol/L x min)	2685.0 (1755.0–4132.5)	2535.0 (1680.0–3577.5)	2700 (1770–4260)	−68.56 (− 287.60, 150.49)	0.551
Insulin iAUC_120min_ (pmol/L x min)	19,215.0 (12,570.0–26,977.5)	17,572.5 (11,700.0–26,505.0)	17,505.0 (13,020.0–27,840.0)	− 309.86 (− 1765.61, 1145.89)	0.408
Fasting GLP1 (pmol/L)	12 (8–16)	12 (9–16)	12 (9–15.5)	− 0.03 (− 0.55, 0.49)	0.846
GLP1 30 min (pmol/L)	27 (19–41)	27 (19–38)	28 (20–38.5)	− 1.16 (− 3.59, 1.28)	0.562
GLP1 120 min (pmol/L)	20 (14–27)	19 (14–27)	21 (16–25)	0.15 (− 1.05, 1.36)	0.989
GLP1 AUC_30min_, pmol/L x min	615 (450–825)	600 (435–795)	645 (450–810)	− 0.87 (− 3.06, 1.37)	0.443
GLP1 AUC_120min_ (pmol/L x min)	2775 (2070–3825)	2715 (1980–3667.5)	2835 (2175–3825)	− 0.62 (− 2.65, 1.44)	0.551
GLP1 iAUC_30min_ (pmol/L x min)	225 (120–420)	210 (120–360)	225 (120–405)	− 17.25 (− 52.33, 17.83)	0.946
GLP1 iAUC_120min_ (pmol/L x min)	1305 (735–2310)	1200 (750–2077.5)	1387.5 (810–2055)	−59.67 (− 230.38, 111.04)	0.929
Fasting GIP (pmol/L)	9 (7–12)	9 (6–12)	8 (6–11)	−0.60 (− 1.16, − 0.04)	0.041
GIP 30 min (pmol/L)	51 (38–66)	47 (36–64)	48 (36–61)	− 1.96 (− 4.10, 0.19)	0.044
GIP 120 min (pmol/L)	45 (33–59)	44 (33–59)	45 (32–60)	0.06 (− 1.78, 1.89)	0.958
GIP AUC_30min_ (pmol/L)	900 (697.5–1170)	840 (660–1125)	855 (645–1080)	− 1.77 (− 3.37, − 0.14)	0.033
GIP AUC_120min_ (pmol/L x min)	5280 (4035–6675)	4957.5 (3915–6532.5)	5055 (3960–6345)	− 1.08 (− 2.58, 0.45)	0.165
GIP iAUC_30min_ (pmol/L x min)	615 (435–840)	585 (420–780)	585 (390–750)	−19.21 (− 49.69, 11.27)	0.215
GIP iAUC_120min_ (pmol/L x min)	4050 (2970–5407.5)	3960 (2895–5212.5)	3960 (3000–5025)	−52.62 (− 233.80, 128.57)	0.527
Fasting glucagon (pmol/L)	10 (7–14)	9 (7–13)	9 (6–13.5)	−1.08 (− 1.78, − 0.38)	0.005
Glucagon 30 min (pmol/L)	9 (6–12)	8 (6–11)	8.5 (6.0–12.0)	−0.69 (− 1.37, − 0.02)	0. 056
Glucagon 120 min (pmol/L)	6 (4–8)	5 (4–7)	6 (4–8)	− 0.36 (− 0.79, 0.07)	0.229
Glucagon AUC_30min_ (pmol/L x min)	285 (210–390)	255 (195–360)	270 (195–375)	−2.27 (− 4.21, − 0.29)	0.025
Glucagon AUC_120min_ (pmol/L x min)	930 (690–1335)	855 (630–1185)	915 (630–1215)	− 1.85 (− 3.78, 0.12)	0.066
Glucagon iAUC_30min_ (pmol/L x min)	−15 (−45–0)	− 15 (− 45–15)	0 (− 45–30)	6.32 (0.87, 11.77)	0.011
Glucagon iAUC_120min_ (pmol/L x min)	−255 (− 510 - -67.5)	− 240 (− 442.5 - -45)	− 135 (− 420–30)	57.86 (18.95, 96.76)	0.003

β, 95% and *P* values adjusted for age, sex and BMI. B and 95% is the effect size estimate using untransformed values. The *P* values were obtained from the inverse normal transformed dependent variable incretin traits and independent variable as GRS and covariates. *P* values < 0.05 are in bold. *AUC* Area under the curve. *iAUC* Incremental AUC. Linear regression analyses adjusted for age, sex, body mass index and the first three genome-wide principal components. AUC_30min_ and AUC_120min_ were log transformed and beta values given in percentage

## Discussion

In this study, we demonstrate that common variants in *hERG*, the minor A-allele of rs36210421 (R1047L) and the minor G-allele of rs1805123 (K897 T), have pleotropic effects, which not only cause shortening of QT-interval, but also alterations in pancreatic and gut hormone release among carriers. We report that the minor A- allele of rs36210421 is associated with increased GLP-1 and decreased GIP response to oral glucose stimulation, whereas the minor G-allele of rs1805123 is associated with decreased fasting plasma insulin and glucagon release. Furthermore, we demonstrate that the additive effect of both variants cause increased GIP secretion and suppressed glucagon secretion, suggesting that hERG potassium channels play an important role in regulation of incretin and pancreatic hormone release.

We report that the minor A-allele of rs36210421 and the minor G-allele of rs1805123 were associated with significantly shorter QT interval among healthy individuals, indicating a mild gain-of-function of both variants in cardiac muscle cells, supporting the findings of previous studies [19, 20, 23, 24]. The hERG ion channel is formed by the KCNH2 protein that consists of six transmembrane alpha helices, a pore helix and cytoplasmically located N- and C-termini. The pore helix is thought to act as a voltage-sensitive sensor [5]. The helices are formed by bindings between amino acids and it is possible that amino acids changes (caused by rs1805123 or rs36210421) could lead to altered folding of the pore helix causing alterations in sensing or in opening/closing of the channel.

*Hylten-Cavallius* et al. recently reported that rare loss-of-function mutations in *hERG* cause prolonged QT-interval and increased GIP and GLP-1 secretion during an OGTT [9]. We report that the two examined gain-of-function *hERG* variants are associated with decreased fasting GIP and GIP secretion during an OGTT, supporting a role of hERG in GIP release from K-cells.

Furthermore, we assessed the effect of the variants in *hERG* on glucose-stimulated insulin secretion. We report that non-diabetic carriers of common variants in *hERG* have lower fasting levels of insulin, but no alterations in glucose-stimulated insulin release during an OGTT in the ADDITION-PRO study. We did not replicate lower fasting insulin levels among carriers of *hERG* variants in the Inter99 study. Thus further studies are needed to clarify whether common *hERG* variants have a role in beta cell function.

The directionality of effect of the common gain-of-function variants in *hERG* seems to be clear in K-cells causing gain-of-function effect leading to decreased secretion of GIP. It is more challenging to assess the effect of the common variants in *hERG* on the alpha-cells in pancreas*.* Alpha-cells secrete glucagon secondary to action potential firing and elevation of cytoplasmic Ca^2+^ concentration [29]**.** These are regulated by fluctuations in glucose and nutrient levels, and perhaps by autocrine and paracrine control by insulin, GABA and Zn^2+^ secreted from adjacent beta-cells and in particular by somatostatin from the delta cells [30–32]. It is challenging to deduce how the common variants in *hERG* influence all these factors. Moreover, the variants in *hERG* may very well have diverging effects in alpha-cells compared to K-cells.

In our study, we found lower fasting glucagon levels and smaller decrements in glucose-stimulated glucagon secretion in hERG gain-of-function carriers. The carriers did, however, have normal glucose levels at fasting and during oral glucose stimulation*.* Blocking of hERG channels in α-cells has been shown to decrease glucagon secretion [6]. We therefore expected that the common gain-of-function variants would cause alpha cells to increase glucagon secretion. Carriers of the gain-of-function variants had lower fasting levels of glucagon, but this might be a consequence of the interplay with insulin and plasma glucose. The mean glucagon levels during OGTT (at 30 and 120 min) were not different from non-carriers, indicating that the suppression of glucagon during glucose-stimulation was probably intact. Supporting this hypothesis, Hardy et al. previously demonstrated that at low glucose levels, blockage of the hERG channel yields a initially stimulatory effect, but then depolarizes the alpha-cells sufficiently to limit action potential firing and as a consequence less glucagon is secreted [6]. hERG channels may therefore be important regulators of glucagon secretion, but detailed in vitro functional studies and/or hyperglycemic clamp studies with arginine stimulation in carriers of common gain-of-function *hERG* variants are needed to pinpoint the exact role of hERG channels in glucagon secretion.

## Conclusions

We demonstrate that common amino acid polymorphisms in the hERG (Kv11.1) voltage-gated potassium channel lead to considerable alterations in plasma GIP and glucagon release, suggesting that voltage-gated hERG potassium channels may play an important role in the regulation of the release of incretin and pancreatic hormones.

## Additional file

Additional file 1: Calculations of AUC and iAUC. Information on how AUC and iAUC was calculated in the cohorts. **Table S1:** Participant characteristics in the ADDITION-PRO cohort and Inter99. Anthropometric measures of individuals in the ADDITION-PRO and Inter99 cohort. **Table S2.** Association of variants (rs36210421 and rs1805123) with QT interval and with metabolic and incretin levels in the Inter99 cohort (*n* = 5487). Table with measures of QTcF interval and glucose levels in the Inter99 cohort sorted by genetic variant and combined in a genetic risk score. **Table S3**. Association of KCNH2 variant rs36210421 with metabolic and incretin levels in ADDITION-PRO cohort (*N* = 1324) (Non diabetes and newly diagnosed T2D). Table with measures of glucose, insulin GIP, GLP-1 and glucagon levels according to carrier status for rs1805123 and rs36210421. **Figure S1.** Levels of incretins and glucagon according to number of risk alleles. Levels of GLP-1, GIP and Glucagon during an OGTT according to number of risk alleles in the GRS. (DOCX 122 kb)

## Abbreviations

AUC: Area under the curve
GIP: Gastric inhibitory polypeptide
GLP-1: Glucagon-like peptide-1
GRS: Genetic risk score
hERG: Human ether-a-go-go channel
*hERG*: Human ether-a-go-go gene
iAUC: Incremental area under the curve
LQTS: Long QT syndrome
OGTT: Oral glucose tolerance test
SNP: Single nucleotide polymorphism
