# Supplementary material for: Common variants in the hERG (KCNH2) voltage-gated potassium channel are associated with altered fasting and glucose-stimulated plasma incretin and glucagon responses
Source: BMC Genet. 2018 Mar 16;19:15. doi: 10.1186/s12863-018-0602-2 (PMC5857134; doi:10.1186/s12863-018-0602-2)
Supplement: Supplementary file 1 — Calculations of AUC and iAUC. Information on how AUC and iAUC was calculated in the cohorts. Table S1: Participant characteristics in the ADDITION-PRO cohort and Inter99. Anthropometric measures of individuals in the ADDITION-PRO and Inter99 cohort. Table S2. Association of variants (rs36210421 and rs1805123) with QT interval and with metabolic and incretin levels in the Inter99 cohort (n = 5487). Table with measures of QTcF interval and glucose levels in the Inter99 cohort sorted by genetic variant and combined in a genetic risk score. Table S3. Association of KCNH2 variant rs36210421 with metabolic and incretin levels in ADDITION-PRO cohort (N = 1324) (Non diabetes and newly diagnosed T2D). Table with measures of glucose, insulin GIP, GLP-1 and glucagon levels according to carrier status for rs1805123 and rs36210421. Figure S1. Levels of incretins and glucagon according to number of risk alleles. Levels of GLP-1, GIP and Glucagon during an OGTT according to number of risk alleles in the GRS. (DOCX 122 kb) [file 12863_2018_602_MOESM1_ESM.docx]

**The common gene variants in the *hERG* voltage-gated potassium channel are associated with altered plasma incretin response and glucagon release**

Line Engelbrechtsen^1,2*^, Yuvaraj Mahendran^1,2*^, Anna Jonsson^1^, Anette Gjesing Prior^1^, Peter E. Weeke^4^, Marit E Jørgensen^5,6^, Kristine Færch^6^, Daniel R Witte^7^, Jens Juul Holst^1,3^, Torben Jørgensen^8,9,10^, Niels Grarup^1^, Oluf Pedersen^1^, Henrik Vestergaard^1,6^, Signe Torekov^1,3^, Jørgen K. Kanters^1,3^,Torben Hansen^1^

***Calculations:***

The total area under the curve (AUC) and incremental /decremental area under the curve (iAUC) from the basal state to 30 and 120 min for GLP1, GIP and glucagon were calculated using trapezoid rule. AUC 0-30min was calculated as [(30 min levels -fasting levels ) x 30 x 1/2 + fasting levels x 30] and AUC 0-120min as [(30 min levels -fasting levels ) x 30 x 1/2 + fasting levels x 30 + 120min levels -30min levels x 90 x ½ + ( 30min levels x 90)]. iAUC 0-30 min was calculated as [(30 min levels -fasting levels ) x 30 x 1/2 + fasting levels x 30 – (fasting levels x 30)]. iAUC 0-120min / iAUC 0-120min as [(30 min levels -fasting levels ) x 30 x 1/2 + fasting levels x 30 + 120min levels -30min levels x 90 x ½ + ( 30min levels x 90) – (fasting level x 120)].

**Table 1: Participant characteristics in the ADDITION-PRO cohort and Inter99**

|  | **ADDITION-PRO** | | **Inter99** | |
| --- | --- | --- | --- | --- |
| Variable | Mean (SD) | Range | Mean (SD) | Range |
| N, Male/female | 1329, 695/634 | - | 5487,2715/2772 | - |
| Age, years | 66.3 (6.9) | 45.2 - 79.8 | 46.2 (7.9) | 29.7 - 61.4 |
| Height, cm | 170.6 (9.1) | 144.5 - 198 | 172.5 (9.2) | 129 - 207 |
| Body mass index, kg/m^2^ | 27.1 (4.6) | 16.0 - 57.9 | 26.2 (4.5) | 15.89 - 56.9 |
| Waist circumference, cm | 95.5 (13.2) | 62.8 – 151.5 | 86.5 (13.2) | 53 - 180 |
| Fasting plasma glucose, mmol/L | 6.0 (0.8) | 2.9 - 12.4 | 5.5 (0.8) | 2.5 - 20.9 |
| Fasting serum insulin, pmol/L | 45.5 (33.9) | 3.0 - 523.0 | 41.92 (27.7) | 5 - 295 |

**Table 2: Association of variants (rs36210421 and rs1805123) with QT interval and with metabolic and incretin levels in the Inter99 cohort (n=** **5487)**

|  | **rs36210421 (MAF=0.031) C>A** | | **rs1805123 (MAF=0.22) T>G** | | | **GRS** | | |
| --- | --- | --- | --- | --- | --- | --- | --- | --- |
| **Trait** | **CC(n=5142)** | **CA(n=341) or AA(n=4)** | **TT(n=3351)** | **TG(n=1843)** | **GG(n=293)** | **0 (n=3091)** | **1 (n=2015)** | **2 (n=380) or 3(n=1)** |
| QTcF (ms) | 416.3(403.7-428.3) | 413.4( 401.7-425.8) | 416.9 (404.9-429.1) | 415.0 (402.2-427.4) | 412.7 (399.8-424.9) | 417.3 (405.2-429.2) | 414.6 (402.1- 427.4) | 413.1 (400.6 -425.1) |
| Glucose 0min, mmol/L | 5.4 (5.1-5.8) | 5.5(5.1-5.8) | 5.4 (5.1-5.8) | 5.4(5.1-5.8) | 5.4(5.2-5.8) | 5.4(5.1-5.8) | 5.5(5.1-5.8) | 5.4(5.2-5.8) |
| Glucose 30min, mmol/L | 8.5 (7.4-9.8) | 8.7(7.5-9.9) | 8.5(7.4-9.8) | 8.5(7.5-9.8) | 8.7(7.5-9.7) | 8.5(7.4-9.8) | 8.6(7.45-9.90) | 8.6(7.5-9.8) |
| Glucose 120min, mmol/L | 5.8 (4.9-7.0) | 5.90(4.9-7.05) | 5.8 (4.9- 7.0) | 5.90 (4.95- 6.90) | 5.80(4.8-6.95) | 5.8(4.9-7.0) | 5.9(4.9-6.9) | 5.8(4.9-7.0) |
| Insulin 0min, pmol/L | 34 (24-51) | 35.0(25-50) | 34 (24-51) | 34 (24 -51) | 36 (23 – 51) | 34(24-51) | 35(24-51) | 35(24-51) |
| Insulin 30min, pmol/L | 244 (175-352) | 263(175-361.5) | 246 (175 - 355) | 244 (174 - 349) | 241.5 (181 – 349) | 245 (175 -354) | 246 (172 -349) | 247 (183-357) |
| Insulin 120min, pmol/L | 155 (95-251) | 161.5(105-274.5) | 155 (95 – 258) | 156 (98 – 245) | 147 (88 – 223.5) | 155 (95-257) | 155 (97 -247) | 158 (95-230) |

QTcF interval measured in milliseconds. GRS is the additive effect of number of risk alleles from both SNPs on variables.

**Table 3: Association of KCNH2 variant rs36210421 with metabolic and incretin levels in ADDITION-PRO cohort (N=1,324) (Non diabetes and newly diagnosed T2D)**

| Trait | **rs36210421 (R1047L) MAF (A)=0.034 C>A** | | | | **rs1805123 ( K897T) MAF(G)=0.24 T>G** | | | | |
| --- | --- | --- | --- | --- | --- | --- | --- | --- | --- |
|  | **Median (inter- quartile range)** | |  |  | **Median (inter- quartile range)** | | |  |  |
|  | **CC (n=1235)** | **CA (n=88) or AA (n=1)** | **Beta (95% CI)** | **P** | **TT (n=777)** | **TG (n=475)** | **GG (n=72)** | **Beta (95% CI)** | **P** |
| Fasting Glucose (mmol/L) | 5.90  (5.56-6.37) | 5.86  (5.46-6.37) | -0.05 (-0.21, 0.11) | 0.76 | 5.90  (5.56-6.37) | 5.90  (5.56-6.37) | 5.96(5.56-6.50) | 0.006 (-0.06, 0.07) | 0.71 |
| Glucose 30min (mmol/L) | 9.12(8.1-10.2) | 9.12(8.30-10.20) | 0.02 (-0.34, 0.39) | 0.79 | 9.22(8.1-10.3) | 9.01(7.99-10.03) | 9.16(8.05-10.19) | -0.11 (-0.26, 0.04) | 0.16 |
| Glucose 120 min (mmol/L) | 6.37(5.25-7.89) | 6.27(5.25-7.29) | -0.13 (-0.61, 0.34) | 0.70 | 6.47(5.25-7.89) | 6.27(5.25-7.65) | 6.34(5.28-8.10) | -0.05 (-0.24, 0.15) | 0.92 |
| Glucose AUC_30min_ | 226.26(204.89-246.00) | 225.63(207.47-246.09) | -0.45 (-7.61, 6.70) | 0.97 | 227.78 (205.50 -247.62) | 223.21(204.89 -243.04) | 228.55(202.61-247.55) | -1.48 (-4.47, 1.51) | 0.37 |
| Glucose AUC_120min_ | 921.82(825.00-1049.99) | 931.73(828.41-1014.44) | -5.33 (-43.42, 32.77) | 0.98 | 925.18(828.73-1051.52) | 914.19(819.58-1028.25) | 935.04(819.28-1077.46) | -8.39 (-24.33, 7.55) | 0.32 |
| Glucose iAUC_30min_ | 46.50 (35.09-60.00) | 47.65(35.55-61.04) | 1.19 (-3.13, 5.52) | 0.54 | 48.00(36.62-61.04) | 44.25(32.05-58.50) | 45.02(35.09-57.22) | -1.78 (-3.58, 0.03) | 0.05 |
| Glucose iAUC_120min_ | 213.63(135.81-305.19) | 211.04(147.25-297.00) | 1.03 (-27.44, 29.49) | 0.78 | 219.74(141.00-306.71) | 204.48(125.13-293.74) | 217.11(121.31-313.91) | -9.235 (-21.14, 2.67) | 0.14 |
| Fasting Insulin (pmol/L) | 37(25-56) | 34(24-51) | -4.53 (-10.95, 1.89) | 0.14 | 38(25-58) | 36(25.5-50) | 39(21.5-59) | -3.2 (-5.9, -0.5) | **0.03** |
| Insulin 30min | 217(147-321) | 215(158.5-282) | 4.39 (-33.89, 42.67) | 0.76 | 223.5(153-329) | 211(144-295) | 234.5(137-338.5) | -9.746 (-25.76, 6.27) | 0.17 |
| Insulin 120min | 187(112-314) | 167(97.5-301.5) | -19.87 (-73.44, 33.70) | 0.28 | 189(113.5-324) | 176.5(106-299) | 217(114.5-344) | -1.47 (-23.87, 20.92) | 0.81 |
| Insulin AUC_30min_ | 3855.0(2632.5 -5632.5) | 3825.0(2827.5 -5010.0) | -0.50 (-619.6, 618.60) | 0.94 | 3960.0(2722.5 -5745.0) | 3735.0(2625.0 -5250.0) | 3945(2475-5820) | -187.84 (-446.83, 71.15) | 0.12 |
| Insulin AUC_120min_ | 23137.5(15645.0 -33345.0 ) | 21465(14925 -32400) | -694.96 (-4680.0, 3290.1) | 0.67 | 23325.0(16035.0- 33592.5) | 21795(15195-32415) | 22575.0(16597.5 - 37882.5) | -647.01 (-2316.09, 1022.08) | 0.26 |
| Insulin iAUC_30min_ | 2640.0 (1698.0 -3990.0) | 2662.5(1860.0- 3577.5) | 135.16 (-408.1, 678.46) | 0.46 | 2685(1770 -4095) | 2535.0(1665.0 -3630.0) | 2700(1728-4275) | -100.94 (-328.35, 126.47) | 0.33 |
| Insulin iAUC_120min_ | 18502.5(12105 – 26955) | 17070(11925-24960) | -151.46 (-3758.2, 3455.3) | 0.84 | 19020 (12420-26940) | 17520.0 (11850.0 -26797.5) | 17715.0(12630.0 -27645.0) | -299.95 (-1810.78, 1210.88) | 0.42 |
| Fasting GLP1 (pmol/L) | 12(9-16) | 11(8-16) | -0.27 (-1.6, 1.02) | 0.85 | 12(8-16) | 12(9-16) | 12(9-16) | 0.03 (-0.51, 0.57) | 0.96 |
| GLP1 30min (pmol/L) | 27(19 -40) | 28.5(18.5 -45.5) | 4.13 (-1.94, 10.20) | 0.36 | 27(19 -41) | 27(19-37) | 28.0(19.5-38.5) | -1.99 (-4.51, 0.53) | 0.31 |
| GLP1 120min (pmol/L) | 20(14-27) | 19(13- 25) | 0.69 (-2.32, 3.69) | 0.49 | 20(14 -27) | 19(14 -26) | 22(16- 27) | 0.12 (-1.14, 1.37) | 0.68 |
| GLP1 AUC_30min_ | 600(450-810) | 630(435-862.5) | 1.335 (-4.19, 7.17) | 0.64 | 615(450-840) | 600(450-780) | 607.5(442.5-802.5) | -1.17 (-3.43, 1.15) | 0.32 |
| GLP1 AUC_120min_ | 2745(2040-3765) | 2775(2025-3937.5) | 1.216 (-3.86, 6.56) | 0.65 | 2775(2070-3855) | 2745(1987.5-3615) | 2812.5(2227.5-3810) | -0.86 (-2.95, 1.27) | 0.43 |
| GLP1 iAUC_30min_ | 210 (120- 405) | 270(142.5- 390) | 72.4 (-15.32, 160.02) | **0.05** | 225(120- 420) | 195(120- 360) | 225(120- 405) | -31.51 (-67.72, 4.71) | 0.35 |
| GLP1 AUC_120min_ | 1245(735 -2250) | 1485(840-1950) | 350.2 (-75.9, 776.4) | 0.08 | 1320(750- 2295) | 1140(720- 2077.5) | 1372.5(855- 2272.5) | -124.09 (-300.41, 52.23) | 0.50 |
| Fasting GIP (pmol/L) | 9 (6.5-12) | 8 (7-11) | -0.71 (-2.10, 0.69) | 0.49 | 9(7-12) | 9(6-12) | 9(6-11) | -0.49 (-1.07, 0.09) | 0.09 |
| GIP 30min (pmol/L) | 49 (37-65) | 50.5 (36.5- 59) | -1.93 (-7.3, 3.39) | 0.57 | 51(38 -65) | 47(36 – 64) | 48.0(36.5 - 62.0) | -1.77 (-4.0, 0.45) | 0.06 |
| GIP 120min (pmol/L) | 45(33 -59) | 39(29 - 57) | -4.34 (-8.89, 0.20) | **0.05** | 45(32 -59) | 44(33 - 57) | 50(35-63) | 0.95 (-0.95, 2.86) | 0.30 |
| GIP AUC_30min_ | 870(675-1155) | 885(660-1050) | -0.94 (-4.91, 3.19) | 0.65 | 900(690-1155) | 840(645-1140) | 855(652.5-1095) | -1.689 (-3.35, -0.00) | **0.05** |
| GIP AUC_120min_ | 5130(4020-6630) | 4965(3892.5-6165) | - 2.026 (-5.673, 1.761) | 0.29 | 5257.5(4035-6630) | 4942.5(3915-6585) | 5137.5(4121.3-6360) | -0.733 (-0.023, 0.850) | 0.36 |
| GIP iAUC_30min_ | 600(435- 810) | 615(427.5- 750) | -14.2 (-89.8, 61.4) | 0.97 | 615(435- 825) | 570(420- 787.5) | 577.5(390- 757.5) | -18.84 (-50.33, 12.65) | 0.18 |
| GIP iAUC_120min_ | 4020(2940- 5385) | 3885(2917.5-4830) | -214.2 (-662.6, 234.3) | 0.42 | 4050(2955- 5385) | 3960(2895-5220) | 4016.25(3081.5-5265) | -17.07 (-204.20, 170.05) | 0.78 |
| Fasting glucagon (pmol/L) | 10(7-14) | 10(8-13) | -0.16 (-1.91, 1.59) | 0.63 | 10(7-14) | 9(7-13) | 9(6-14) | -1.0 (-1.5, -0.4) | **0.003** |
| Glucagon 30min (pmol/L) | 8(6-12) | 9(6-11.5) | 0.51 (-1.18, 2.19) | 0.43 | 9(6-12) | 8(6-11) | 9(6-12) | -0.67 (-1.2, -0.13) | **0.02** |
| Glucagon 120min (pmol/L) | 6(4-8) | 5(4-7) | -0.12 (-1.18, 0.95) | 0.81 | 6(4-8) | 5(4-7) | 6(4-8) | -0.29 (-0.67, 0.10) | 0.27 |
| Glucagon AUC_30min_ | 270(195-375) | 285.0(217.5-360) | 1.878 (-3.10, 7.12) | 0.47 | 285(210-390) | 255(180-360) | 255(195-382.5) | -2.732 (-4.73, -0.70) | **0.009** |
| Glucagon AUC_120min_ | 900(660-1260) | 930.0(697.5-1140) | 1.588 (-3.33, 6.76) | 0.53 | 930(690-1320) | 855(630-1185) | 915(630-1222.5) | -2.266 (-4.25, -0.24) | **0.03** |
| Glucagon iAUC_30min_ | -15(-45 - 15) | -15(-45 - 15) | 10.65 (-2.98, 24.28) | 0.17 | -15(-45 – 7.5) | -15(-45 - 0) | 0(-45 - 30) | 4.64 (-1.01, 10.28) | **0.04** |
| Glucagon iAUC_120min_ | -240(-480 - -45) | -240(-450 - -90) | 46.24 (-51.05, 143.53) | 0.79 | -255(-510 - -75) | -240(-435 - -45) | -105(-420 - 90) | 48.37 (9.57, 87.18) | **0.006** |

β, 95% and P values adjusted for age, sex, BMI and the first three PCAs. β and 95% is the effect size estimate using untransformed values. The P values were obtained from the inverse normal transformed dependent variable incretin traits and independent variable as GRS and covariates. P values < 0.05 are in bold. AUC: Area under the curve; iAUC: Incremental area under the curve. AUC_30min_ and AUC_120min_ were log transformed and Beta values given in percentage.

**Figure 1. Levels of incretins and glucagon according to number of risk alleles.**
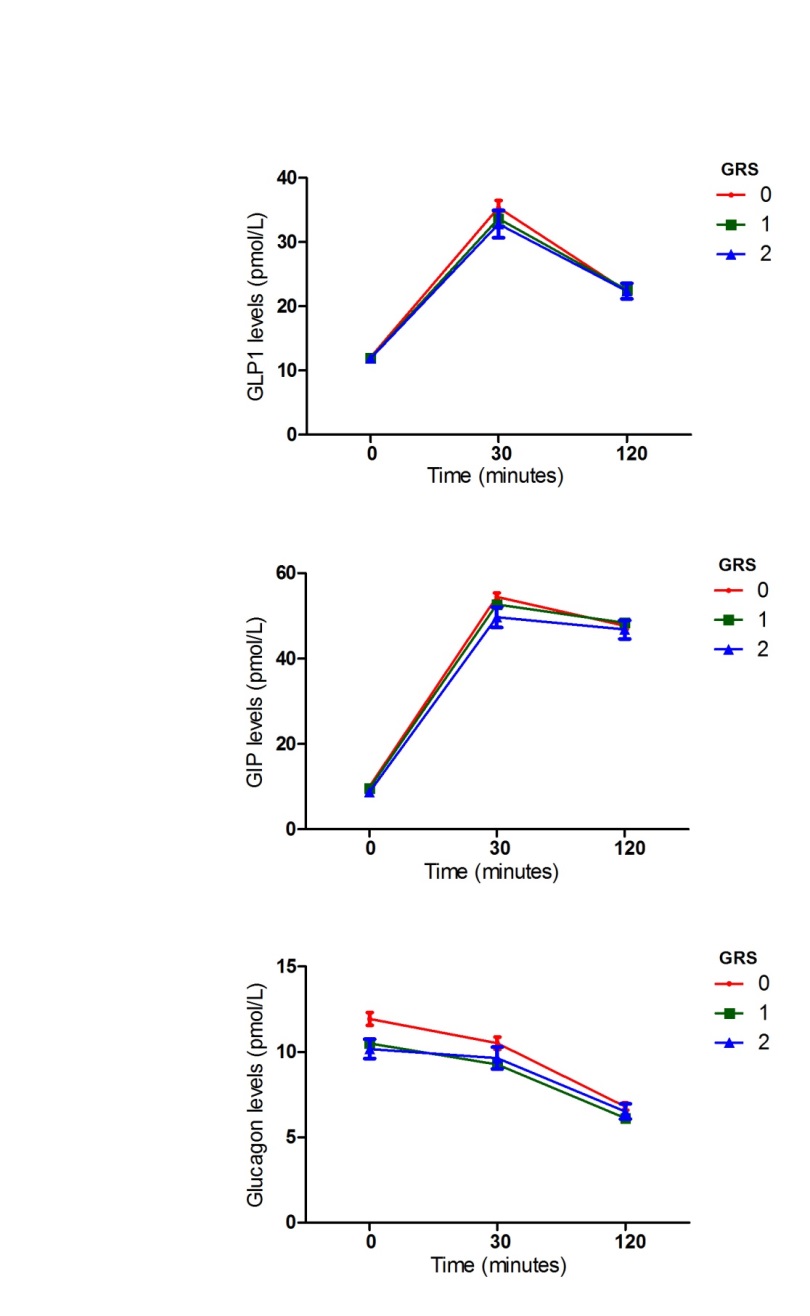


Mean and SEM of incretin and glucagon levels in fasting, 30 min, 120 min according to genetic risk score (GRS) from (rs36210421 and rs1805123). GRS=0 indicates no risk alleles, GRS=1 indicates carriers of one risk allele of either rs1805123 or rs36210421, GRS=2 indicates two or more risk alleles of either rs1805123 or rs36210421.
